# Hyperreflexia and Preserved Reflexes in Pediatric Guillain-Barré Syndrome: A Case Report and Literature Review

**DOI:** 10.7759/cureus.85172

**Published:** 2025-06-01

**Authors:** Shahd Abuhalima, Dania Abuhalima, Abdulkareem Saymeh, Maysa Alawneh, Issa Alawneh

**Affiliations:** 1 Department of Medicine, An-Najah National University, Nablus, PSE; 2 Department of Pediatrics, An-Najah National University, Nablus, PSE; 3 Department of Pediatrics and Neonatology, An-Najah National University, Nablus, PSE; 4 Department of Pediatric Neurology, An-Najah National University, Nablus, PSE

**Keywords:** acute motor axonal neuropathy, aman, atypical presentation, guillain-barré syndrome, hyperreflexia

## Abstract

Guillain-Barré syndrome (GBS) is the most prevalent cause of acute flaccid paralysis, often following an antecedent illness such as upper respiratory infections. Acute motor axonal neuropathy (AMAN), a subtype of GBS, predominantly affects motor axons, resulting in significant motor impairment, and is frequently associated with *Campylobacter jejuni* infection. We present a case of a three-year-old female patient who developed AMAN, a subtype of Guillain-Barré Syndrome (GBS). Approximately 10 days prior to admission, she had a history of fever, sore throat, and cough, which slightly improved for one day following a five-day course of antibiotics. However, her condition then worsened, and she began experiencing vomiting of gastric contents and occasional sputum production. Despite initial improvement, her condition deteriorated, leading to decreased muscle strength, exaggerated reflexes, and progressive respiratory failure. Neurophysiological studies, including nerve conduction studies (NCSs) and imaging, were consistent with AMAN. The patient was treated with intravenous immunoglobulin (IVIG) therapy and required endotracheal intubation for respiratory support. Over the course of her hospitalization, her neurological status improved significantly, and by day 9, she was able to walk with assistance, marking a successful recovery. This case highlights the importance of early diagnosis and intervention in managing GBS in pediatric patients, particularly in the context of atypical presentations, including hyperreflexia.

## Introduction

Guillain-Barré syndrome (GBS) is the most common cause of acute flaccid paralysis worldwide. Most patients develop progressive motor weakness following an antecedent illness, commonly an upper respiratory tract infection [1]. Acute motor axonal neuropathy (AMAN) is a subtype of GBS characterized by selective involvement of motor axons, often triggered by *Campylobacter jejuni* infection [2]. AMAN often leads to significant motor impairment, with electrophysiological studies showing reduced compound muscle action potentials (CMAPs), which are measures of electrical activity in muscles following nerve stimulation. Some patients may present with atypical signs such as normal or increased tendon reflexes (hyperreflexia) early in the disease course. Bulbar involvement, affecting muscles that control swallowing and speech, can also occur.

The prevalence of AMAN among pediatric GBS cases varies by geographic region; for example, a study from North India reported AMAN in up to 69.4% of pediatric GBS cases, underscoring its significant presence in certain populations [2]. AMAN tends to be more common in children than adults and is generally associated with a worse prognosis compared to demyelinating forms of GBS.

The following case report describes a previously healthy toddler who developed AMAN with atypical clinical features, illustrating these challenges in diagnosis and management.

## Case presentation

A previously healthy three-year-old female patient presented with a 10-day history of fever, sore throat, and mild cough. Her symptoms initially improved for one day after a course of antibiotics, but then progressed to vomiting gastric contents and occasional sputum production. Despite treatment, her condition worsened over the next five days. Three days prior to admission, she exhibited decreased oral intake, reduced activity, increased drowsiness, a hoarse voice, weak cry, and minimal speaking efforts, indicating significant clinical decline.

Our patient initially presented with fever, sore throat, cough, vomiting, hoarse voice, and progressive limb weakness. Notably, early hyperreflexia was observed, an atypical but increasingly recognized initial sign in AMAN.

Her neurological symptoms rapidly progressed in an ascending pattern, beginning with lower limb weakness and advancing to involve the upper limbs and respiratory muscles, ultimately leading to respiratory failure and the need for mechanical ventilation.

Upon admission, the patient was conscious but confused and drowsy. Her Glasgow Coma Scale (GCS) was 14/15. Muscle strength was Medical Research Council (MRC) grade 2/5 in the lower extremities and 4+/5 in the upper extremities. Bilateral foot drop was noted, with the ability to perform plantar flexion but an inability to dorsiflex. The knee-jerk reflexes were exaggerated bilaterally, indicating hyperreflexia, while sensory examination remained normal, with no clonus and an absent Babinski sign bilaterally. Pupils were mid-dilated with sluggish reaction to light.

Cranial nerve assessment revealed bulbar involvement. The patient had a hoarse cry and voice, a weak gag reflex, and minimal vocal effort, suggesting involvement of cranial nerves IX and X. No facial asymmetry, ptosis, or ophthalmoplegia was observed, indicating no apparent involvement of cranial nerves III, IV, VI, or VII.

On the following day, the patient’s condition deteriorated. Neurological examination revealed worsening weakness in both upper and lower extremities, along with increased difficulty breathing. Also, the patient became areflexic, with a positive Babinski sign bilaterally. Bulbar and respiratory muscle involvement, hypercapnia, and a decrease in oxygen saturation levels all prompted the need for endotracheal intubation, and the patient remained intubated for four days. Her diagnosis was most consistent with AMAN, so intravenous immunoglobulin (IVIG) therapy was recommended at 2 g/kg (40 g), divided over two days. Following the initiation of IVIG and subsequent extubation, the patient showed progressive improvement in respiratory function and limb strength over the course of hospitalization. Muscle strength in the lower limbs improved from 2/5 on admission to 3/5 by day 5, reaching 5/5 by discharge. Similarly, upper limb strength improved from 4+/5 to full strength. This progression is detailed in Table 1.

**Table 1:** Neurological status and muscle strength progression during hospitalization GCS: Glasgow Coma Scale

Day of hospitalization	Muscle power (lower limbs)	Muscle power (upper limbs)	Knee-jerk reflexes	Consciousness (GCS)	Functional status (e.g., ambulation)
Admission (day 1)	2/5	4+/5	Hyperreflexia	14/15	Unable to walk
Day 5	3/5	4+/5	Normal	15/15	Improved limb movement
Discharge (day 9)	5/5 (estimated)	5/5 (estimated)	Normal	15/15	Able to walk with assistance

A nerve conduction study (NCS), conducted after initial stabilization, revealed a marked reduction in CMAPs, with preserved sensory nerve action potentials (SNAPs). For example, the CMAP amplitudes in the peroneal and tibial nerves were markedly reduced to less than 1 mV (normal: >4 mV), while distal motor latencies and conduction velocities remained within normal limits. These findings are consistent with the AMAN subtype of GBS, characterized by selective involvement of motor axons without significant demyelination.

Cerebrospinal fluid (CSF) analysis results are presented in Table 2. The protein level was elevated, but the presence of mild pleocytosis and a high red blood cell count likely resulted from a traumatic lumbar puncture. Therefore, the typical albuminocytologic dissociation seen in Guillain-Barré syndrome was not clearly demonstrated. Nevertheless, the clinical presentation and electrophysiological findings supported the diagnosis of AMAN.

**Table 2:** CSF analysis Cerebrospinal fluid analysis showed elevated protein and mild pleocytosis, although the high RBC count suggests a traumatic lumbar puncture. CSF: cerebrospinal fluid, RBC: red blood cell, WBC: white blood cell

Test	Result	Reference range/comment
WBCs	16/µL (75% neutrophils)	0-5/µL (mostly lymphocytes)
RBCs	5,000/µL	Elevated (likely traumatic tap)
Glucose	82 mg/dL	50-80 mg/dL
Protein	385 mg/L	150-450 mg/L

**Figure 1:**
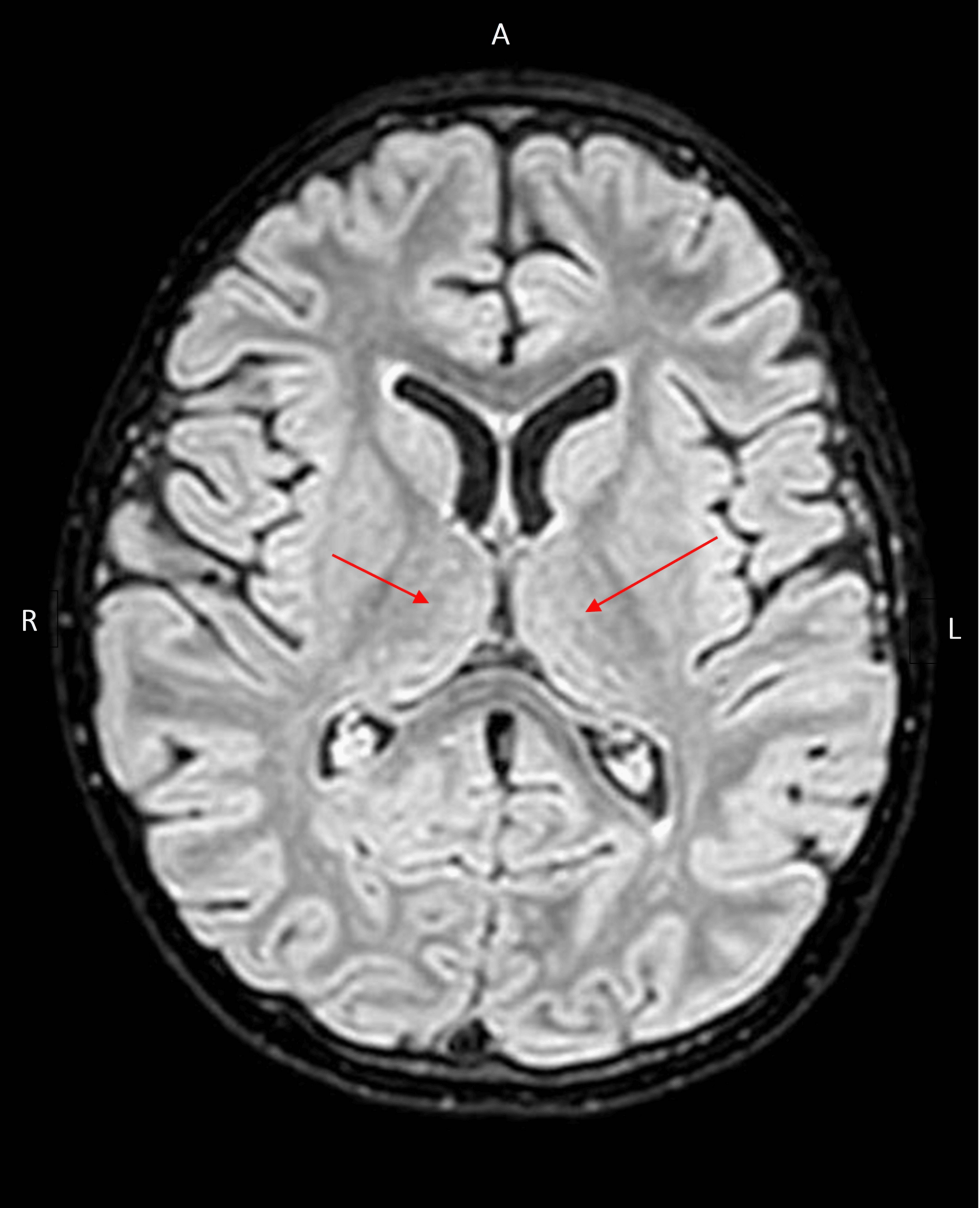
Brain MRI of the patient on admission Axial FLAIR brain MRI showing multiple nonspecific periventricular white matter hyperintense foci (red arrows). No diffusion restriction or abnormal enhancement is seen. The absence of central nervous system lesions helps exclude differential diagnoses such as ADEM, supporting a peripheral etiology consistent with the AMAN subtype of GBS. FLAIR: fluid-attenuated inversion recovery, MRI: magnetic resonance imaging, ADEM: acute disseminated encephalomyelitis, AMAN: acute motor axonal neuropathy, GBS: Guillain-Barré syndrome

Imaging was conducted, including brain and spinal magnetic resonance imaging (MRI). Brain MRI showed nonspecific periventricular white matter hyperintense foci without diffusion restriction or abnormal enhancement, as shown in Figure 1. These findings were considered unremarkable, with no evidence of central demyelination such as that seen in acute disseminated encephalomyelitis (ADEM) or other encephalomyelitides. This supports a diagnosis confined to the peripheral nervous system.

**Figure 2:**
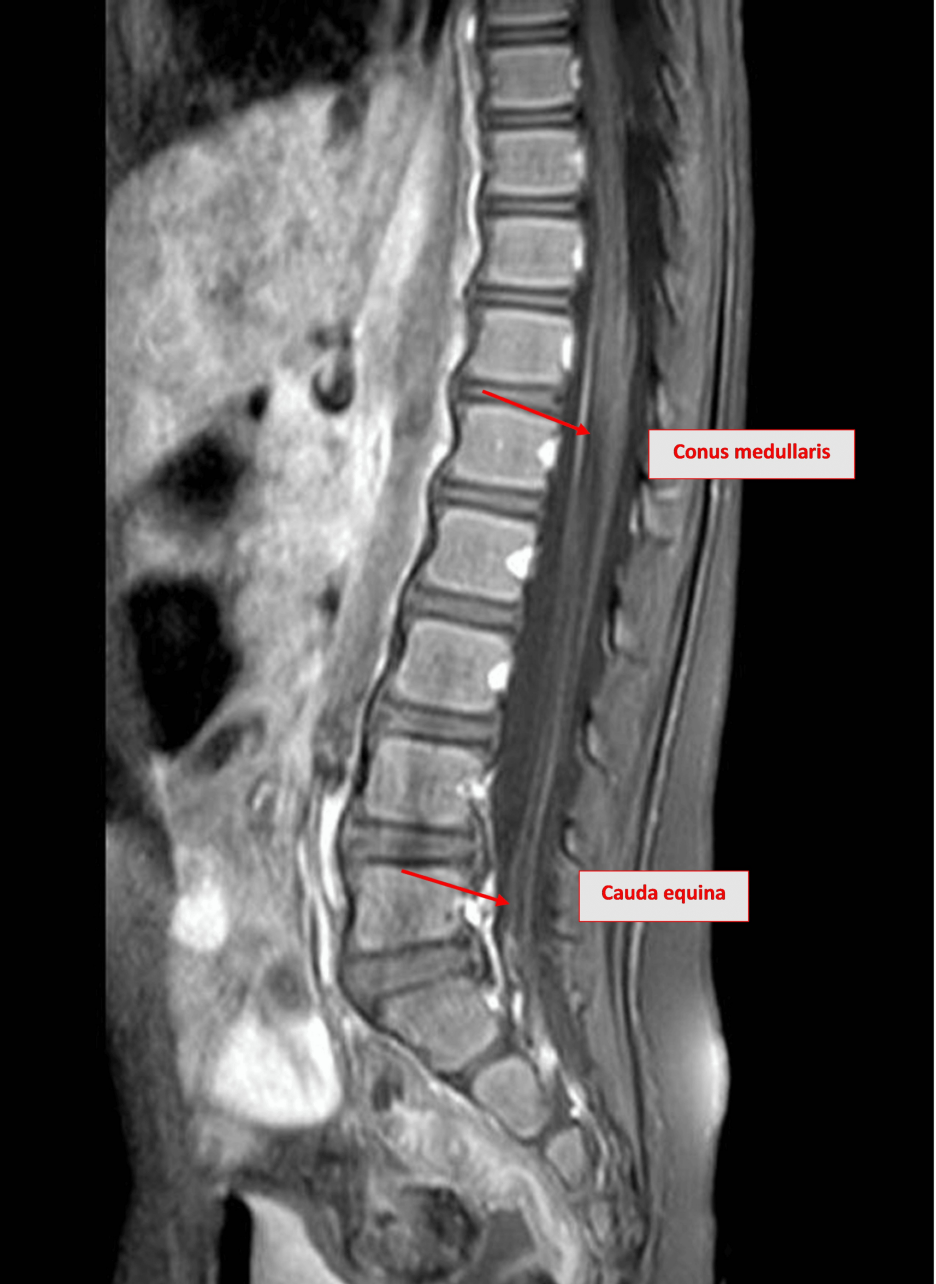
Spinal MRI of the patient on admission T1-weighted MRI of the lumbar spine demonstrating smooth thickening and enhancement of spinal nerve roots within the cauda equina and conus medullaris region (red arrows) in sagittal view, findings consistent with Guillain-Barré syndrome. MRI: magnetic resonance imaging

The cervical, dorsal, and lumbar spine MRIs showed no intrinsic cord lesions, preserved vertebral alignment, and normal caliber of the central canal. However, the lumbar spine MRI revealed smooth thickening and enhancement of the spinal nerve roots in the cauda equina and conus medullaris region, suggesting Guillain-Barré syndrome, as shown in Figure 2.

The findings were correlated with clinical and laboratory results, and the management plan was adjusted accordingly to address the patient’s critical condition. Table 3 shows the initial laboratory results upon presentation.

**Table 3:** Laboratory results upon admission CBC: complete blood count, BUN: blood urea nitrogen, CRP: C-reactive protein, AST: aspartate aminotransferase, ALT: alanine transaminase, LDH: lactate dehydrogenase, CK: creatine kinase

Parameter	Result	Reference range
CBC with differential		
White blood cell count	23 k/µL	4-9 k/µL
Neutrophil	80%	50%-70%
Lymphocyte	14%	18%-42%
Hemoglobin	12.3 g/dL	13.7-17.2 g/dL
Platelets	692 k/µL	140-450 k/µL
BUN	3.9 mg/dL	5-22 mg/dL
Creatinine	0.2 mg/dL	0.7-1.2 mg/dL
Sodium	132.1 mEq/L	135-155 mEq/L
Potassium	3.85 mEq/L	3.5-4.8 mEq/L
Chloride	93.4 mEq/L	98-107 mEq/L
Magnesium, serum	2.14 mg/dL	1.6-2.6 mg/dL
Phosphate, serum	4.05 mg/dL	2.5-4.5 mg/dL
Prothrombin time	13.8 seconds	11-14 seconds
International normalized ratio	1.07	0.8-1.2 (unitless)
Activated partial thromboplastin time	26.9 seconds	25-40 seconds
CRP (quantitative)	8 mg/L	0-5 mg/L
AST	23 U/L	5-40 U/L
ALT	10.5 U/L	7-56 U/L
Albumin	4.63 g/dL	3.5-5.0 g/dL
Alkaline phosphatase	150 U/L	44-147 U/L
Total serum bilirubin	0.18 mg/dL	0.1-1.2 mg/dL
Fibrinogen	378 mg/dL	200-400 mg/dL
LDH	267 U/L	140-280 U/L
Ferritin	89.5 ng/mL	20-300 ng/mL
CK	25 U/L	22-198 U/L

Differential diagnoses such as transverse myelitis, myasthenia gravis, and botulism were considered. However, normal MRI findings, absence of fatigable weakness or cranial nerve involvement, and the clinical progression all made these less likely. The electrophysiological findings and response to IVIG supported a final diagnosis of the AMAN subtype of Guillain-Barré syndrome.

Throughout her hospitalization, the patient demonstrated steady clinical improvement. Her neurological status stabilized, with normalization of knee-jerk reflexes and no clonus observed. Muscle tone remained within normal limits in all limbs, and spontaneous movement was present throughout. She maintained good truncal and head control, reflecting improved functional stability. By discharge on day 9, bedside examination suggested near-complete recovery of muscle strength (estimated MRC grade 5/5), and she was able to walk with assistance, an important milestone in her recovery.

She was referred for outpatient physiotherapy and follow-up with pediatric neurology. At her two-week follow-up visit, she showed continued strength improvement and increasing independence in ambulation.

## Discussion

AMAN is a distinct subtype of GBS characterized by axonal degeneration of motor nerves, often triggered by an antecedent infection. Unlike the more common acute inflammatory demyelinating polyneuropathy (AIDP), AMAN primarily affects the motor axons without significant demyelination, leading to profound weakness with relatively preserved sensory function [3].

In our case, we describe a three-year-old female patient diagnosed with AMAN, who initially presented with hyperreflexia and progressive weakness following a febrile upper respiratory infection. The initial symptoms included limb weakness and difficulty walking, which rapidly worsened. She later developed bulbar involvement, areflexia, and respiratory failure requiring intubation and ICU admission. Her neurological symptoms progressed rapidly in an ascending pattern, beginning in the lower limbs and advancing to involve the respiratory muscles, consistent with the classic GBS presentation.

This case highlights an atypical AMAN presentation, characterized by early hyperreflexia followed by bulbar dysfunction and diaphragmatic involvement, as evidenced by respiratory failure necessitating mechanical ventilation.

Eleven cases of Guillain-Barré syndrome with hyperreflexia or preserved reflexes in the pediatric population were found in the literature. Table 4 summarizes key findings from all cases and compares them with the current reported case.

**Table 4:** Summary of pediatric cases of Guillain-Barré syndrome with hyperreflexia or preserved reflexes in comparison to the current case LL: lower limb, DTR: deep tendon reflex, ICU: intensive care unit, CMAP: compound muscle action potential, SNAP: sensory nerve action potential, AIPN: acute inflammatory polyradiculoneuropathy, IVIG: intravenous immunoglobulin, AMAN: acute motor axonal neuropathy, AMSAN: acute motor sensory axonal neuropathy, GBS: Guillain-Barré syndrome, UL: upper limb, URI: upper respiratory tract infection, MRI: magnetic resonance imaging

Case	Age, sex	Initial presentation	Reflexes (DTRs)	ICU stay	Bulbar involvement	Nerve conduction study	Treatment	Diagnosis	Prognosis	Time to recovery	Preceding illness	MRI result
Current case	3, female	Fever, sore throat, cough, vomiting, hoarse voice, weakness	Exaggerated knee jerks bilaterally	Yes, intubated for 4 days	Hoarse voice, weak cry, respiratory distress	↓ CMAPs, preserved SNAPs	IVIG 2 g/kg, respiratory support	AMAN	Good, full cognitive recovery	Full recovery by day 9	URI+ fever 10 days before, brief improvement, then gastroenteritis	Nerve root thickening (cauda equina/conus medullaris), lung consolidation
Case 1 [4]	17, male	LL weakness, pain, gait difficulty (10 days)	Asymmetric LL reflexes (L normal, R ↓)	No	No	↓ CMAP amplitudes, normal conduction velocities, no block, normal somatosensory conduction	IVIG 0.4 g/kg/day × 4	AMAN	Gradual resolution	Not mentioned	Febrile gastroenteritis 14 days before	Mild thickening of the upper lumbar roots
Case 2 [4]	10, male	Wrist/palm weakness (1 month), difficulty writing	Asymmetric LL reflexes (R absent, L normal)	No	No	↓ CMAP amplitudes (peroneal/median), mild slowing, no block, normal somatosensory conduction	No treatment	AMSAN	Gradual improvement	2 months	No infection	Normal thoracic MRI
Case 3 [5]	14, male	Vision loss, numbness, limb weakness	Brisk all limbs	No	No	↓ CMAP amplitudes, absent SNAPs, preserved velocities	IVIG 0.4 g/kg/day × 5	AMSAN/Miller-Fisher variant	Recovered over weeks	Not mentioned	URI 10 days prior	Not reported
Case 4 [6]	5, male	LL pain, inability to walk/sit up	Initially 3+ LL reflexes → ↓	No	Loss of gag reflex	↓ CMAP (peroneal), normal velocities, no block, absent sural somatosensory response	IVIG 2 g/kg × 3	AMSAN/Miller-Fisher variant	UL strength returned first	Discharged after 2 weeks (walking with support)	Fever + rash 7 days before	Normal MRI
Case 5 [7]	1 year 11 months, male	Ascending paralysis (2 weeks)	Hyperreflexia (3+), cross adductors +	No	No	↓ Median/tibial motor amplitudes	None	AMAN	Slow recovery, walking with support	8 weeks, (hyperreflexia persisted)	URI 4 weeks before	Normal MRI
Case 6 [8]	10, male	Asymmetric limb/shoulder weakness	Preserved	No	Bilateral facial palsy, loss of voice	Motor axonal polyneuropathy	IVIG and IV steroids	Motor axonal polyneuropathy	Gradual resolution	2 weeks	URI 10 days before	No MRI
Case 7 [9]	12, female	Unsteady gait, hands/feet numbness	Brisk	No	Bilateral lateral gaze palsy, uvula deviation, gag loss	Absent SNAPs, ↓ motor amplitudes, no slowing	IVIG 2 g/kg	AMSAN	Strength 5/5 at 8 months, brisk DTRs at 18 months	Not reported	Acute gastroenteritis (1 week)	Not reported
Case 8 [10]	9, female	Limb weakness (13 days)	Brisk	No	No	↓ CMAP, slight slowing, peroneal unrecordable	IVIG 400 mg/kg/day × 5	Motor axonal polyneuropathy	Gradual recovery to grade 4+	6 weeks	Sore throat 6-7 days before	Normal MRI
Case 9 [11]	11, male	Facial diplegia, pain (head, abdomen, thigh)	Preserved/hyper reflexive	No	Bilateral VII nerve paresis, reduced palate/tongue movement	Abnormal motor/sensory, transient UL weakness	IVIG 0.4 g/kg/day × 5	Atypical GBS (post-Arepanrix vaccine)	Improved strength/gait	Discharged on day 5	H1N1 vaccine 13 days prior	Normal MRI
Case 10 [12]	8, male	Slurred speech, diplopia	Normal	Not mentioned, ventilation required	Weak gag, tongue/palate weakness	Normal, except ↓ peroneal velocity (43 m/s)	Not mentioned	AIPN	Strength returned	3 months	URI 1 week prior, bee sting 5 days prior	No MRI
Case 11 [12]	17, male	Nasal speech, fluid regurgitation	Normal	No	Absent gag reflex, no voluntary palate function	Normal conduction, prolonged latency	Not mentioned	AIPN	Full recovery	1 month	Bee sting 3 days prior, no infection	No MRI

Among the reported pediatric cases, male patients slightly outnumbered female patients (7 out of 11), and the age range varied from under two years to 17 years, highlighting that this presentation spans the pediatric age spectrum.

Several key features distinguish our case. First, the early presence of hyperreflexia is an uncommon finding in AMAN but has been documented in certain cases of GBS with pure motor involvement (Cases 3, 4, 5, 7, 8, and 9).

The rapid clinical deterioration leading to respiratory failure underscores the potential severity of AMAN in pediatric patients, necessitating prompt recognition and intensive care support, which is similar to Case 11.

Bulbar involvement in AMAN is uncommon but has been reported in several cases [13]. In our review, Cases 4, 6, 7, 9, 10, and 11 exhibited varying degrees of bulbar dysfunction, including dysphagia, dysarthria, and respiratory compromise. Notably, Case 10 required mechanical ventilation, emphasizing the potential severity of bulbar dysfunction in pediatric AMAN. Similarly, our case of AMAN demonstrated progressive bulbar involvement, leading to respiratory failure and the need for intubation. This highlights the importance of early recognition of bulbar symptoms, as they can precede or parallel respiratory decline, necessitating close monitoring and early airway intervention.

While bulbar involvement is more commonly associated with AIDP [14], its presence in AMAN suggests a possible overlap in pathophysiological mechanisms or a subset of AMAN cases with greater cranial nerve involvement [15]. Further studies are needed to determine whether bulbar dysfunction in AMAN correlates with specific antibody profiles, more severe disease courses, or prolonged recovery times [16].

Triggering factors were identified in most cases. Upper respiratory tract infections were the most common (Cases 3, 6, 8, and 10), followed by gastrointestinal infections (Cases 1 and 7). Less typical triggers included vaccination (Case 9) and a bee sting (Case 10). The free interval between the trigger and neurological symptoms ranged from three to 14 days in most cases, but none exceeded one month, aligning with our current case, where symptoms began 10 days after a flu-like syndrome.

We noticed that most cases had a preceding illness before the onset of AMAN. Upper respiratory tract infections were reported in Cases 3, 6, 8, and 10, while gastroenteritis was documented in Cases 1 and 7. However, some cases had less typical triggers, such as Case 9, which developed symptoms 13 days after receiving the Arepanrix H1N1 vaccine, and Case 10, which was associated with a bee sting three days before symptom onset. Although fever was a common associated symptom, the interval between the triggering event and disease onset varied among cases, but none exceeded one month. In our current case, the patient had flu-like symptoms 10 days before developing neurological symptoms, aligning with previous reports of gastrointestinal infections as potential triggers of AMAN.

These findings highlight the diversity of antecedent events in AMAN, suggesting that while infections are the most common triggers, immune-mediated responses to other stimuli, such as vaccines and insect stings, may also contribute to disease onset.

MRI findings of smooth thickening and enhancement of spinal nerve roots in the cauda equina and conus medullaris region provide additional evidence supporting the diagnosis, aligning with reports of similar radiologic findings in GBS-related neuropathies (Case 1), which suggests a possible imaging pattern in certain AMAN cases. The periventricular white matter hyperintense foci observed on MRI in our case were deemed incidental and not typical for AMAN, which usually does not involve central nervous system lesions. These findings likely represent nonspecific or post-viral changes and are considered unrelated to the patient’s acute peripheral neuropathy.

Not all cases had detailed nerve conduction study (NCS) findings reported. However, among those who did, the majority demonstrated low CMAP amplitudes, including Cases 1, 2, 3, 4, 5, 7, and 8, indicating motor axonal degeneration. Case 6 exhibited a motor axonal polyneuropathy pattern, which is characteristic of AMAN. In contrast, Cases 10 and 11 had normal NCS findings. Interestingly, Case 7 had absent sensory nerve action potentials (SNAPs) in the extremities, while Case 9 displayed abnormal sensory findings. In our case, the nerve conduction study performed after initial stabilization revealed a marked reduction in CMAPs with preserved SNAPs, consistent with the diagnosis of the AMAN subtype of GBS.

These findings reinforce the heterogeneous nature of GBS in different forms of the disease, where most cases align with a typical motor axonal pattern, but occasional cases demonstrate sensory involvement or even normal NCS results. This could underscore the importance of timing in electrophysiological testing and the potential need for repeat NCS evaluations to track disease progression and recovery.

In terms of diagnosis, the majority of patients, including our case, were diagnosed with AMAN or acute motor sensory axonal neuropathy (AMSAN). Specifically, our case was diagnosed as AMAN. Similarly, Cases 1 and 5 were diagnosed with AMAN, Cases 6 and 8 were diagnosed with motor axonal polyneuropathy, while Cases 2, 3, 4, and 7 were diagnosed with AMSAN. Additionally, a few cases presented with variants of these conditions. Cases 3 and 4 were diagnosed with AMSAN, while also exhibiting features of the Miller-Fisher variant. Case 5 was diagnosed with AMAN but had a slow recovery over an extended period, with hyperreflexia persisting. Other rare diagnoses included Case 9, which was identified as an atypical Guillain-Barré syndrome post-vaccination. On the other hand, Cases 10 and 11 were diagnosed with acute inflammatory polyradiculoneuropathy (AIPN). These cases highlight the variability in diagnostic classification within the broader spectrum of acute motor neuropathies.

Regarding treatment, the current patient showed a remarkable response to IVIG administered over two days. When comparing our management approach with the literature, we found that Cases 1, 3, 4, 6, 7, 8, and 9 also received IVIG, with varying degrees of clinical improvement. Additionally, Case 5 was treated with prednisolone, suggesting that corticosteroids were considered in certain cases, although their role in AMAN remains controversial.

AMAN is typically associated with a poorer prognosis, but early IVIG treatment may mitigate axonal damage and improve outcomes. The general trend in pediatric AMAN cases supports IVIG as the primary treatment, with early administration potentially influencing recovery outcomes [17]. Corticosteroids are not routinely recommended for AMAN or other GBS variants, as evidence suggests they do not improve outcomes and may even delay recovery [18]. Our case further reinforces that timely IVIG administration can significantly alter disease progression and improve outcomes [19].

In terms of prognosis, most patients in the reviewed cases had a favorable outcome, with many experiencing significant recovery, often within a few weeks. For instance, in our case, the patient achieved full recovery by day 9, with no lasting cognitive or motor deficits. Similarly, Case 1, diagnosed with AMAN, showed gradual resolution with no long-term complications, while Case 8, also diagnosed with AMAN, improved to grade 4+ strength in six weeks. In contrast, some cases took longer to recover, such as Case 5, where the patient’s recovery was slow, taking about eight weeks, with hyperreflexia still present at the end of the recovery period. Several patients in the AMSAN group, including Cases 4 and 7, had a more gradual improvement over several months, although they did not experience permanent impairments. Overall, the prognosis appeared generally good, with most patients showing full or near-full recovery, although the duration of recovery varied depending on the severity of symptoms and treatment response.

Prognostic factors such as the Erasmus GBS outcome score, which incorporates clinical features including age, preceding diarrhea, and severity of muscle weakness, could provide valuable insight into predicting recovery outcomes. While this score was not specifically calculated in our case, the early initiation of IVIG and rapid clinical improvement align with favorable prognostic indicators suggested by the Erasmus model.

Regarding recovery time, our case demonstrated a prolonged but steady improvement following diagnosis and treatment, achieving full recovery by day 9. When comparing our case to others in the table, we found that Case 10 exhibited a similarly rapid recovery, being discharged by day 5. Cases 1, 3, and 4 showed gradual resolution of symptoms, with Case 4 requiring two weeks for supported ambulation. In contrast, Case 5 experienced a significantly prolonged recovery over eight weeks, while Case 10 required three months for full-strength restoration. The variability in recovery time appears to be influenced by factors such as disease severity, early intervention, individual response to treatment, and the rapidity of symptom onset. Early recognition and treatment are crucial in improving outcomes, as delayed intervention may lead to prolonged recovery or residual deficits. Our patient exhibited rapid symptom progression but received early IVIG therapy, which likely contributed to the favorable clinical course observed. These findings align with existing literature suggesting that early recognition and management, particularly the timely administration of IVIG, significantly impact recovery duration [20]. Further analysis is required to determine the precise factors contributing to variations in recovery times among similar cases.

## Conclusions

Although areflexia is a hallmark of GBS, this case highlights that hyperreflexia may be observed in the early or evolving stages, particularly in the AMAN subtype. The diagnosis was supported by nerve conduction studies demonstrating reduced CMAPs without evidence of demyelination, a finding consistent with AMAN. Despite the initial presentation with brisk reflexes, the patient responded well to IVIG, achieving near-complete motor recovery by discharge and remaining symptom-free at short-term follow-up. This case underscores the importance of recognizing atypical presentations of pediatric GBS and emphasizes the role of early diagnosis and treatment in improving outcomes.
